# Enacting Phenomenological Gestalts in Ultra-Trail Running: An Inductive Analysis of Trail Runners’ Courses of Experience

**DOI:** 10.3389/fpsyg.2018.02038

**Published:** 2018-10-26

**Authors:** Nadège Rochat, Vincent Gesbert, Ludovic Seifert, Denis Hauw

**Affiliations:** ^1^Center for the Study and the Transformation of Physical Activities (CETAPS EA 3832), Faculty of Sport Sciences, University of Rouen Normandy, Mont-Saint-Aignan, France; ^2^Centre de Recherche en Psychologie de la Santé, du Sport et du Vieillissement (PHASE), Institute of Sport Sciences of the University of Lausanne (ISSUL), Lausanne, Switzerland; ^3^Raidlight-Vertical Outdoor Lab Company, Saint-Pierre-de-Chartreuse, France

**Keywords:** enaction, phenomenology, gestalts, experience, trail running

## Abstract

Using an enactive approach to trail runners’ activity, this study sought to identify and characterize runners’ phenomenological gestalts, which are forms of experience that synthesize the heterogeneous sensorimotor, cognitive and emotional information that emerges in race situations. By an in-depth examination of their meaningful experiences, we were able to highlight the different typologies of interactions between bodily processes (e.g., sensations and pains), behaviors (e.g., actions and strategies), and environment (e.g., meteorological conditions and route profile). Ten non-professional runners who ran an ultra-trail running race (330 km, 24,000 m of elevation gain) volunteered to participate in the study. Data were collected in two steps: (1) collection of past activity traces (i.e., race maps, field notes, and self-assessment scales) and (2) enactive interviews using the past activity traces in which the runners were invited to relive their experience and describe their activity. The enactive interviews were coded using the course-of-experience methodology to identify the phenomenological gestalts that emerged from activity and scaffolded the runners’ courses of experience. The results revealed that runners typically enact three phenomenological gestalts: controlling global ease, enduring general fatigue and experiencing difficult situations, and feeling freedom in the running pace. These phenomenological gestalts were made up of specific behaviors, involvements, and meaningful situated elements that portrayed various ways of achieving an ultra-endurance performance in the race situation. They also highlighted how runners enact a meaningful world by acting in relation to the fluctuations in physical sensations and environmental conditions during an ultra-trail race. Practical applications for preparation, race management and sports psychology interventions are proposed to enrich the existing recommendations. In conclusion, this approach provides new research perspectives by offering a more holistic grasp of activity in trail running through an in-depth analysis of athletes’ experience. In doing so, we may expect that runners can connect these typical gestalts to their own personal experiences and stories as trail runners in order to sustain a viable approach to their sport.

## Introduction

Ultra-trail running is an endurance sport that consists of running and walking self-sufficiently on hiking trails in a natural and changing environment over distances from 80 to more than 300 km (International Trailrunning Association, 2013), with aid stations usually set up by the race organizers along the route. This ultra-endurance discipline is extremely challenging, and runners must deal with environmental and meteorological conditions, altitude changes, the topography of the terrain, the onset of pain and fatigue, muscular damage (e.g., Schmidt Easthope et al., 2010), exhaustion and effects due to sleep deprivation (e.g., Hurdiel et al., 2015; Poussel et al., 2015). Furthermore, Nicolas et al. (2011) showed that runners’ perceptions of stress after a 24-h ultra-marathon alter their psychological recovery. Not only does this sport require high commitment in terms of preparation and strategy, but it also enables runners to pursue personal development and discovery by pushing their physical and mental boundaries (Simpson et al., 2014). From this perspective, ultra-trail running undeniably constitutes a fascinating area for research in sports psychology, as witnessed by a growing number of contributions to the scientific literature (see Roebuck et al., 2018, for a systematic review).

Within the sports psychology literature, three well-defined research axes have been identified, and they have all produced interesting – yet fragmented – insights into the accomplishment of ultra-endurance performances. The first research axis investigated ultra-endurance runners’ personality profiles and cognitive orientations. For example, Acevedo et al. (1992) characterized the cognitive orientations of ultra-marathoners who completed a 100-mile trail run (160 km) and showed that these athletes had unique sport-specific cognitive orientations, as they were more confident and committed to running, displaying slightly more competitiveness and personal performance orientation (i.e., high commitment to their personal time goals), with little concern for win/loss outcome (i.e., lower commitment to ranking goals) in comparison to athletes from other sports. In the same vein, Hughes et al. (2003) investigated runners’ personality profiles by combining personality traits (i.e., neuroticism, extraversion, openness, agreeableness, and conscientiousness) and sensation seeking indicators (i.e., thrill and adventure seeking, experience seeking, disinhibiting, and boredom susceptibility) to determine whether these were predicting factors of participation. The authors showed that runners displayed common personality traits, such as higher scores on extraversion and openness than control groups, without, however, considering them as causal factors of participation in this type of challenge.

The second research axis mainly investigated the changes and monitoring of emotions and profiles of mood states (POMS). For example, Tharion et al. (1988) investigated the changes in ultra-marathon runners’ POMS. The pre- and post-race comparisons showed that after the race, runners had significantly less tension and vigor and more depression, confusion and fatigue. These results suggest that running an ultra-marathon significantly impacts five of the six mood factors (i.e., with the exception of anger). Lane et al. (2004) investigated the evolution of mood responses during athletic performances in extreme environments (i.e., altitude and/or extreme temperatures) and showed that POMS are potentially a relevant indicator of negative adaptations to the environment. Other studies specifically analyzed emotions in ultra-endurance sports; for example, Lane and Wilson (2011) investigated ultra-marathoners’ emotional intelligence and showed that runners were able to efficiently regulate their emotional states in order to delay the emergence of negative emotions to reach optimal performance. Last, Stanley et al. (2012) identified five strategies that runners use to regulate their emotions before the race. These consisted of (1) task preparation (e.g., listening to music and visualization), (2) avoidance (e.g., distraction and downplaying outcomes), (3) positive thinking (e.g., recalling past performance accomplishments and anticipating pleasant emotions after running), (4) negative thinking (e.g., negative focus and anticipating unpleasant emotions after running), and (5) self in relation to others (e.g., receiving or giving social support). However, the effectiveness of these strategies and their emergence in the many situations runners encounter remain unclear. In addition, it should be noted that the role of embodied and contextual elements – although constitutive of human performance – seemed to have been put aside in the above-mentioned works.

The third axis consists of *in situ* analyses via a descriptive phenomenological approach, which focuses on runners’ experience unfolding during a race, in order to track changes in experience over time. For example, Holt et al. (2014) provided insights into how runners overcome stressors (i.e., injuries, cramping, gastrointestinal problems, or thoughts about quitting) by using coping strategies (i.e., setting short-term goals, monitoring pace, hydration and nutrition, mental battle, and social support). In addition, by clustering meaningful units in ultra-marathon runners’ experiences, a study conducted in existential phenomenology highlighted the major themes that emerge from the runners’ experiences during ultra-distance races, such as preparation and strategy, management, discovery, personal achievement, and community (Simpson et al., 2014). In the continuity of these above-mentioned results, several studies were conducted using the enactive paradigm to analyze trail runners’ activity in race situations. Characterizing runners’ activity with an enactive approach enables researchers to grasp the dynamics of embodied experiences through in-depth analysis of the continuous interactions between agents and their environment (Varela et al., 1991; McGann et al., 2013). The phenomenological connection with the enactive approach was outlined by Thompson (2010): Conceptually, the enactive approach considers the human body as being fundamentally a “lived body” (p. 16), which directly stems from Merleau-Ponty’s existentialist phenomenology. As an illustration of the conceptual continuum between the enactive approach and phenomenology, Rochat et al. (2017) analyzed the phenomenological states of trail race finishers and withdrawers, assuming that these states reflected vitality during the race. Vitality was defined as a subjective variable, which refers to the psychological experience of possessing aliveness and energy (Ryan and Frederick, 1997; Akin et al., 2016). They found that runners typically go through three vitality states: states of vitality preservation, vitality loss, and vitality revival. The distribution of these states revealed that finishers experienced significantly more vitality preservation, while withdrawers experienced significantly more vitality loss, which impacted the race outcome (i.e., finish vs. withdraw). Furthermore, the temporal organization of these states, and particularly the cumulative effect of the succession of these vitality states, showed that (1) ensuring vitality preservation is a central point for finishing a race and (2) the race outcome (i.e., finish or withdraw) begins to take shape relatively early and is sensitive to the runners’ initial vitality states. In the same vein, Antonini Philippe et al. (2016) provided a detailed account of withdrawers’ activity by identifying the stages in runners’ courses of experience leading up to withdrawal (i.e., feeling pain, putting meaning to those feelings, adjusting one’s running style, attempting to overcome the problem, other runners’ influences, assessing the situation, and deciding to withdraw). In addition, Hauw et al. (2017) articulated experiential data and behavioral data (i.e., runners’ elevation velocities) during an ultra-trail race to characterize typical activity patterns. They identified three activity profiles: economical runners (i.e., who maintain slow running speeds throughout the entire race in order to preserve themselves as much as possible and who display a progressive decrease in elevation velocity), explorer runners (i.e., who alternate between states of ease, during which they accelerate, and states of extreme fatigue, with these changes associated with high running speed at the beginning of the race followed by substantial decreases in elevation velocity), and “Kairos” runners (i.e., who maintain a slow pace during a big part of the race and then accelerate for the last kilometers, thus displaying stable velocity followed by an abrupt increase in elevation velocity). Taken together, these studies, which highlighted the relevance of the enactive approach to analyzing runners’ activity, showed how runners interacted with the many situations during an ultra-trail race and how this is revealed at the phenomenological level of experience.

However, despite the insights into the meaningful parts that make up the stream of runners’ experiences, evidence is sparse about their activity, which encompasses behaviors, intentions, interactions with the environment, and the significations that runners enact. As these runners must cope with the above-mentioned physical and environmental factors, their ongoing activity gives rise to the phenomenological experience of the relationship between activity and the situation in which it occurs. From this perspective, activity can be considered through the lens of the enactive approach and its underlying core ideas, which are the constituents of the guiding theory adopted for the present study (i.e., the 4Es of embodied, extended, embedded and enacted) (Rowlands, 2010). The first core idea is that activity is fundamentally Embedded in the environment: the interactions between the agent’s activity and the environment define a meaningful situation that is relevant for the agent (McGann et al., 2013). Therefore, action and situation (as meaningfully enacted by the agent) are co-determined and do not stem from a pre-existing mental representation or appraisal. The second core idea is that activity is Embodied, which means that the body is a key element for organizing activity by synthesizing the ongoing sensorimotor processes (Varela et al., 1991; Gibbs, 2005; Froese and Fuchs, 2012). The idea of embodiment refers to the bodily anchorage of lived experience, as the interactions between an actor and the environment are primarily based on a bodily engagement. This means that the brain, the body and the world are indivisible and have reciprocal relationships, contrasting with the view of the traditional cognitive sciences, which assume that the body is controlled by the brain (Di Paolo et al., 2011). The third core idea suggests that activity is Extended in the sense that agents may use the situation and artifacts to act beyond their body to manipulate and exploit external structures and hence extend their ranges of possible actions (Rowlands, 2010). Last, the fourth core idea is that agents Enact significations from their activity and situation by a process of sense-making (Di Paolo et al., 2011; Froese and Di Paolo, 2011), which refers to the generation of meaning enacted by agents during their activity. Meaning emerges from the coupling between the meaninful situation and the agent’s activity. From an enactive perspective, this idea of sense-making is crucial for understanding activity because it suggests that agents enact a meaningful world in which they articulate significations and ways of acting that emerge at the level of experience (Stewart et al., 2010). As Thompson (2005) suggested, experience should be the object of phenomenological analysis since the enactive approach assumes that the meaning that a person gives to what he/she is doing, feeling or thinking does not come from an external realm but is enacted in the interaction between agents and the situation.

Phenomenology can be defined as the analysis of how a phenomenon appears in consciousness though the body in movement (Davidsen, 2013), suggesting that the dynamics of this experienced motion over time takes the form of an experience scheme that can be globalized into phenomenological gestalts to characterize structures of activities (Lakoff and Johnson, 1980). A gestalt is a form of experience in which the heterogeneous sensorimotor information is synthesized by grouping together significant units of experience (Rosenthal and Visetti, 1999; Tversky et al., 2008). Lakoff and Johnson (1980) used the term “experiential gestalts” and defined them as follows: “An experiential gestalt is a multidimensional structured whole arising naturally within experience.” In this sense, gestalts are strictly linked to phenomenology because their identification implies the study of individuals’ experience that can be grasped through verbal descriptions to identify the fundamental characteristics (Rosenthal and Visetti, 1999). These organizational structures of experience are transposable throughout many situations and, even though they may vary, they can re-build global experience (Lakoff and Johnson, 1980) as sequences of key moments (Tversky et al., 2008). In other words, gestalts synthesize different aspects of experience such that movement, perception, feelings, cognitions, and emotions are not fragmented but appear as a coherent and meaningful whole in experience. Therefore, analyzing phenomenological experiences should allow us to identify runners’ gestalts, which are macroscopic forms of experience in relation to the performance emerging during a trail running race. In addition, Rosenthal and Visetti (1999) argued that instead of breaking down a phenomenon to identify the causal processes or isolated factors that explain phenomena, a phenomenological gestalt approach seeks to investigate the organization of activity as a whole that contains sense and unity - neither of which is divisible. In addition, the notion of gestalt is congruent with the enactive approach in the sense that activity is not prescribed by mental representations or motor schemes, but rather is understood as a co-determination through activity and situation (Stewart et al., 2010).

We postulate that characterizing the gestalts that scaffold runners’ experience will provide insights into how runners make sense of their activity and situation so as to act effectively and finish an extreme race. Ultra-trail running has increasingly attracted recreational runners (Hoffman et al., 2010) who tend to train autonomously (Krouse et al., 2011), and it therefore seems timely and important to enhance our knowledge on what happens during a race (i.e., what processes are at stake when runners achieve their performances). In this way, the added value of an in-depth investigation of runners’ meaningful experiences is that we do not reduce their activity and performance to a single psychological process, but instead we highlight the different typologies of interactions between bodily processes (e.g., sensations and pains), behaviors (e.g., actions and strategies), and environment (e.g., meteorological conditions and route profile) under the form of phenomenological gestalts. We therefore analyzed runners’ embedded activity throughout a race with two aims: (1) to identify common phenomenological characteristics that can be defined as phenomenological gestalts and (2) to analyze in depth the content of these gestalts in order to provide a detailed account on the trail runners’ meaningful activity in the race situation. In sum, by qualitatively documenting the embedded activity, we expected to gain insight into how ultra-trail runners are able to turn in such extreme performances, thus opening up perspectives to enrich the existing practical recommendations for training, physical and mental preparation, injury prevention, and performance.

## Materials and Methods

### Research Design

Stemming from the enactive paradigm, the course-of-action framework provides the methodological tools for grasping the phenomenological gestalts that emerge from embedded activity (Theureau, 2006). This framework analyzes, for example, runners’ embedded activity through the examination of their experience using retrospective interviews and is defined as follows: “the activity of a given actor engaged in a given physical and social environment, where the activity is meaningful for that actor; that is, he can show it, tell it and comment upon it to an observer-listener at any instant during its unfolding” (Theureau and Jeffroy, 1994, p. 19). To grasp this meaningful part of activity, we will focus on the pre-reflexive level of consciousness that emerges from the activity-environment couplings, which means that the actor can show, mime, simulate, tell about and comment on his/her activity at any time during its occurrence to an observer-interlocutor in favorable conditions (Theureau, 2006). The favorable conditions refer to the specific actions that the researcher undertakes (these will be outlined in the data analysis section) during the interviews to help the interviewee adopt a “phenomenological attitude” (Thompson, 2010), which means: “Stepping back from the natural attitude, not to deny it, but in order to investigate the very experience it comprises” (p. 18). Therefore, access to pre-reflexive consciousness provides a disclosure of the world as meaningfully experienced by the agent. Furthermore, within the sports psychology literature, the course-of-action framework has already been used to gain insight into performance in many sports like trampoline and acrobatics (Hauw and Durand, 2004, 2008), skydiving (Mohamed et al., 2015), table tennis (Sève et al., 2005), orienteering (Mottet et al., 2016), rowing (Sève et al., 2013), soccer (Villemain and Hauw, 2014; Gesbert et al., 2017), and trail running (Antonini Philippe et al., 2016; Hauw et al., 2017; Rochat et al., 2017). All these studies analyzed embedded activity through activity analysis and were able to provide practical recommendations for training, education, and competition (e.g., Hauw, 2017, 2018). Therefore, for research into athletes’ embedded activity, the course-of-action framework provides the methodological tools to identify the temporal and meaningful structures of experiences.

### Participants

Our sample was composed of 10 runners (5 men and 5 women) participating in the Tor des Géants in Italy (330 km with 24,000 m of elevation gain). They were between 31 and 58 years old (*M* = 42.4, *SD* = 8.66). All had volunteered to participate in the study; they were recruited via a message posted on the Raidlight community forum (i.e., a well-known forum dedicated to the French-speaking community of trail runners) and by snowball sampling. All were non-professional athletes but had experience in ultra-endurance races (*M* = 9 years, *SD* = 2.9) and trained on average 80 km weekly (*SD* = 30). They all expressed a keen interest in participating in the study on condition that the data collection would remain non-invasive during the race.

In total, 660 runners took part in the race and 72% were finishers. The cut-off time to finish the race was set at 150 h. During this event, the meteorological conditions were particularly challenging, with bad weather and low temperatures. Consequently, the race had to be stopped 87 h after the start for safety reasons, hence before all participants had crossed the finish line. Runners who were still racing when the organizers announced this decision were considered finishers. The rankings were established in function of the lap times at the last checkpoint. Only the first six race participants were able to cross the finish line (the winner won in 80 h) and the participants of our sample had covered between 200.3 and 303.2 km during the 87 h of race.

### Data Collection

Data were collected in two steps: First, race data were collected, including race maps (aerial views with aid stations, route, altitudes, and landmarks identified) with a detailed kilometer scale. Field notes were also taken by three researchers and one assistant at the life bases (i.e., refreshment points where runners had the possibility to eat warm meals and sleep) to collect *in situ* data. These field notes contained short observations of the participants’ general states or runners’ spontaneous verbalizations. Race data also included self-assessment scales (see Supplementary Material) that aimed to rate (1) the perceived intensity of the effort (10-point Likert scale), (2) the level of perceived fatigue (7-point Likert scale), (3) the intensity of the perceived muscular damage (7-point Likert scale), and (4) the level of well-being (7-point Likert scale). Runners were asked to complete the scales at the start of the race and at each life base (i.e., six life bases along the route). The collection of race data was designed to interfere as little as possible with the runners’ activity. These data (i.e., race maps, field notes, and self-assessment scales) were used as past activity traces and were not further processed or associated with the results of the present study; their collection and use aimed to restore the full context and chronology of the events during the enactive interviews, as has been done in previous research (e.g., Hauw and Bilard, 2012; Hauw and Mohamed, 2015). They were thus used to re-build the dynamics of personal experience in relation to specific events. This approach was shown to increase the accuracy and insights during the elicitation process in retrospective interviews (Drasch and Matthes, 2013). Regarding the interpersonal interactions between participants and the research team, all participants had met the researchers before the start, so they were familiar with them and thus expected to meet at least one member of the research team at the life bases.

Second, individual enactive interviews (in French) about these past activity traces were conducted between 5 and 7 days after the race. As reported in Rochat et al. (2017), these enactive interviews were designed to provoke the re-enactment of the stream of experience that emerged during the race, and the past activity traces helped the runners relive their meaningful experience. The interview was designed as follows: runners were invited to describe their activity chronologically, while deliberately ignoring the outcome and avoiding post-analysis and retrospective generalizations. In this way, the researchers sought to help them adopt a “phenomenological attitude” (Thompson, 2010) by which they would be able to restore the story of their race, telling us what they did and describing their behaviors, thoughts, concerns, and the elements that were drawing their attention. When needed, the researcher asked questions like: “What were you doing at this given moment?”; “What was your state of mind like at this particular moment?”; “What were you trying to do?”; and “What drew your attention?” in order to collect supplementary information about emotions, concerns, thoughts, and interpretation. The interviews were recorded and transcribed for further analysis. They lasted between 60 and 120 min. The interviews were made by the three researchers who were also present during the race to collect field notes. They were experienced in conducting enactive interviews about performance in several sports, including trail running. They had prior knowledge of trail running without, however, being regular expert runners, and they were thus unfamiliar with the inner culture of ultra-trail running and the common expressions used by trail runners (i.e., trail running slang). Importantly – and in contrast with semi-structured interviews – the enactive interviews were conducted with no prior themes, categories, or assumptions regarding the content of the experience accounts. Nevertheless, the *granularity* of the experience accounts was controlled, which means that the researchers sought to (1) unveil the underlying processes of the runners’ behaviors and cognitions by inviting them to explicit their embodied sensations, concerns, and emotions (Smith and Osborn, 2015), (2) restore the temporal chain of events and their effects on the participants’ experience, and (3) help the runners bracket their actual judgments on their past activity. Practically, to control the granularity, researchers did not hesitate to ask the runners for more information when their explanations were unclear; this helped to make their descriptions of their experience more explicit and detailed as they explained their expressions and the underlying sensations and experience to ensure the accuracy of the information. An example of this is as follows: “So you’re saying that you were feeling pain here, can you describe what you were feeling exactly?” Although we acknowledge that retrospective interviews inherently prompt the interpretation of one’s past experience, these precautions (i.e., the use of past activity traces, the verbal prompts, and the control of the granularity of experience) ensured that we could access runners’ pre-reflexive consciousness as closely as possible.

### Ethics Statement

The protocol was approved by the ethics committees of both the University of Rouen Normandy and the University of Lausanne (joint agreement) and followed the guidelines of the Declaration of Helsinki. Procedures were explained to the participants, who then gave their written informed consent to participate.

### Data Analysis

**FIGURE 1:**
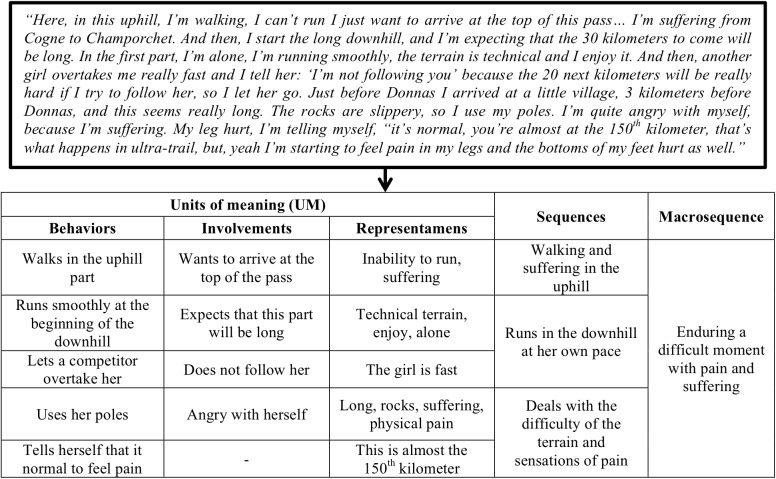
Example of coding from an extract of the verbatim of the enactive interview.

Four researchers (i.e., the three researchers who collected data plus one external researcher) who were familiar with course-of-action theory and methodology coded the enactive interviews. The coding was done in four steps: (1) labeling units of meaning in the courses of experiences, (2) identifying meaningful sequences, (3) identifying and sorting macro sequences, and (4) clustering those sequences that contained the same theme (i.e., similar range of concerns associated with the macro sequences): these clusters were then considered as belonging to the same phenomenological gestalt. For example, the macro sequences in which runners were forced to slow down (e.g., because of injury, fatigue, bad weather, etc.) were grouped together as belonging to a similar gestalt, as opposed to slowdowns to manage a viable pace or to rest, which constituted another theme, hence another gestalt. First, to restore the unfolding race as a chronological story, we identified the elementary units of meaning (EUM). Each EUM corresponds to a single action associated with cognitive features like thoughts, concerns, and interpretation, also coded into categories. The involvement in the activity refers to the possibilities that are conceivable by the actor in the situation; it expresses how the actor enters into activity (Theureau, 2006). Thus, in this study it highlighted the runner’s concerns opening a field of possible actions (Reed, 1993). These concerns enacted the emergence of the actual behaviors and the representamens, which are the elements in the situation that are significant to the actor when the action occurs (e.g., a specific element in the race environment, or physical sensations). Hence, the runners’ behaviors were adapted on the basis of what they did and felt as meaningful. The representamen may come from the environment (e.g., competitors, weather conditions, etc.) or be a sensation (e.g., pain, fatigue, etc.). The relationships among involvements, representamens and action (i.e., what the actor actually does) form the EUM, which is a condensed unity of the enaction process at the level of what a person felt, thought and did. The macrostructures, which are chains of EUMs, give rise to phenomenological gestalts. All these elements were labeled in a table following the race chronology and reporting the stream of EUMs (Figure 1). Each EUM was labeled with an action verb. An important consideration is that the coding must restore the story of the race by sticking to the stream of meaningful action units. To do so, the past activity traces (i.e., race maps and field notes) were used to check and accurately situate the chronology of the meaningful events that emerged in the runners’ activity.

Second, the EUMs were grouped into larger structures called sequences, which provided an overall depiction of the history of the EUM chain by summarizing their content and breaking down the flow of the race (Figure 1). Two EUMs belonged to the same sequence if one was partly determined by the outcome of the previous one or if they both referred to the same theme (i.e., when EUMS contained similar and recurrent representamens and concerns around which behaviors were organized) (Hauw et al., 2003; Hauw and Durand, 2004). The formulation of the sequences should synthesize the EUM contents.

Third, we identified macro sequences, which were made up of the chronological chain of sequences in which activity occurred with the same general dynamics. The macro sequences restored the general story of the runners’ experience by splitting it into chapters, which followed the race chronology. We then identified the typical macro sequences on the basis of a systematic search for similarities in the involvements, actions, and representamens that made up each macro sequence.

#### Characterization of Phenomenological Gestalts

The previous steps enabled us to split the runners’ courses of experience into meaningful structures of experience (i.e., macro sequences). Next, to identify the phenomenological gestalts, we conducted an inductive analysis of the involvements, behaviors, and representamens of all runners’ macro sequences. These were clustered and listed in a recapitulative table, which enabled us to inductively distinguish typical phenomenological gestalts that were common to all runners. When needed, we checked the coding of the stream of EUMs, verbatims and traces to ensure a full understanding of the context that characterized the macro sequence.

### Ensuring Data Reliability

Several precautions were taken to ensure the trustworthiness of the data analysis. As noted, the interviewers carefully followed the interview guide to obtain the same quality of experience accounts. Three trained researchers who were familiar with the coding procedures then coded the contents independently and compared their findings to ensure agreement on the number and label of the EUMs, sequences, and macro sequence. In cases of disagreement, researchers discussed until consensus was reached.

## Results

### Typical Phenomenological Gestalts

In total, 39 macro sequences (MS) were found in the 10 participants’ courses of experience; these were characterized as belonging to three phenomenological gestalts: controlling global ease (17 MS), enduring general fatigue and experiencing difficult situations (13 MS), and feeling freedom in the running pace (9 MS).

#### Controlling Global Ease

**FIGURE 2:**
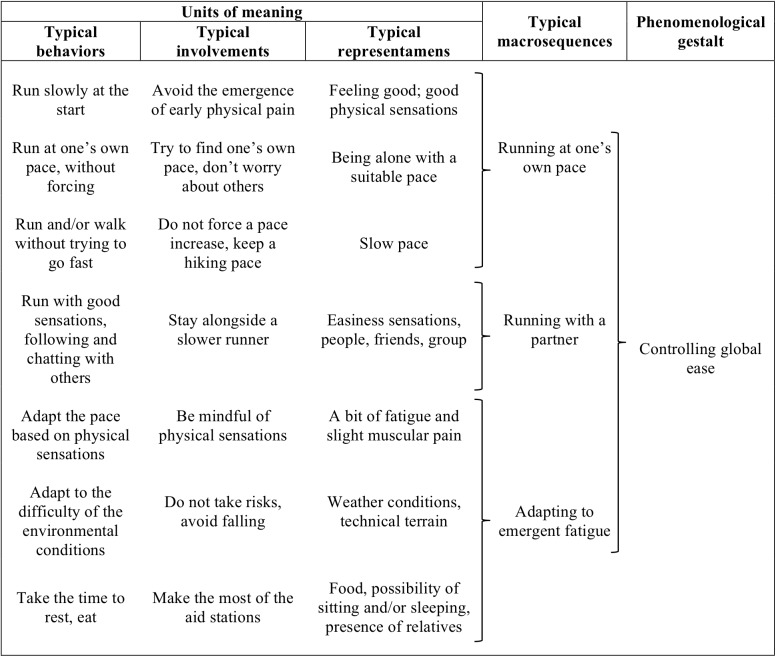
Content of the gestalt “controlling global ease.”

This phenomenological gestalt refers to behaviors aimed at maintaining physical and mental resources in order to ensure full control of the race. Runners were concerned about preserving their physical integrity (i.e., not getting injured) and their energetic resources (i.e., staying in a good state and preventing pain) to finish the race and enjoy the event (Figure 2). More specifically, this gestalt was made up of three typical activities identified in their courses of experience: (1) running with a partner, (2) running at one’s own pace, and (3) adapting to emergent fatigue. When running with a partner, runners ran part of the route while chatting with this person, as the race provides the opportunity to catch up with people during the effort:

“With my partner, we keep to the same cadence for the climb, we feel good, we’re just where we want to be in the group, so we keep the same rhythm as the others and start the climb easily. And there are lots of other runners to talk to.”

When they ran at their own pace, the runners had made the decision to stop adjusting their pace to others and instead focus in on their own physical sensations in order to keep full control of race management:

“I don’t at all feel like it’s a big effort. It’s like I’m out hiking, it’s a hiking rhythm… And then the guy next to me said he wanted to speed up to go fast in the first part of the race and make fewer stops to sleep. I told him I was keeping to my rhythm, nice and easy like I usually do.”

When adapting to emergent fatigue, runners felt slight fatigue and muscular pain, which prompted them to adapt the pace (i.e., slowing down and maintaining an easy speed) and/or took the time for procedures (i.e., eating and resting) at the refreshment points. They also made local adaptations in response to short episodes of emergent fatigue, without, however, breaking the stream of their activity of ensuring their physical integrity:

Runner: “I had a refueling meal and then headed out easily and there I felt that I was a little slow. After the first climb I lay down on the ground and slept for 20 min in the sun. I lay down and then woke up because I was snoring…”
Researcher: “You slept because you got drowsy after eating?”
Runner: “Yes, I got drowsy because I ate. I slept 20 min and woke up. As soon as I woke up, I felt refreshed and I was able to continue the climb.”

#### Enduring General Fatigue and Experiencing Difficult Situations

**FIGURE 3:**
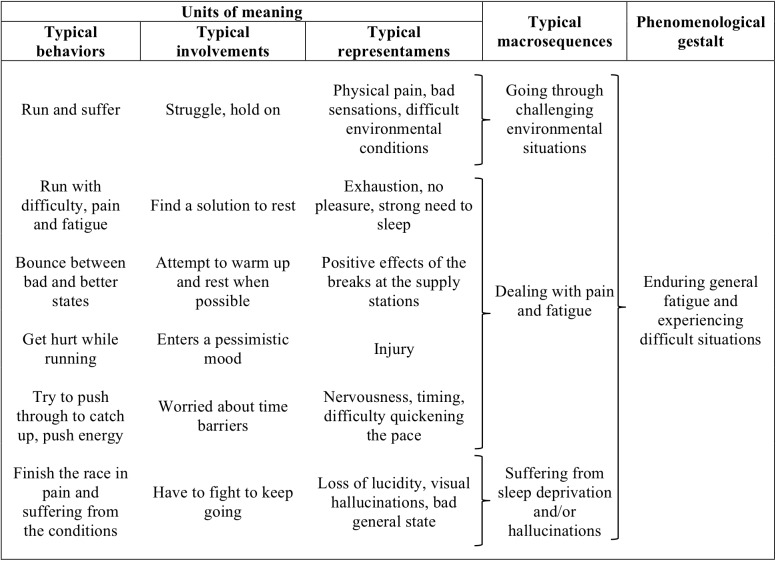
Content of the gestalt: enduring general fatigue and experiencing difficult situations.

This phenomenological gestalt refers to runners’ constrained behaviors because of the emergence of negative representamens (e.g., exhaustion, pain, hallucinations, etc.) and the difficulty of exiting this state, which led to involvements of hanging on to stay in the race despite tough moments (Figure 3). Specifically, the runners had to deal with three typologies of difficult situations: (1) dealing with pain and fatigue, (2) going through challenging environmental conditions, and (3) suffering from sleep deprivation and/or hallucinations. When dealing with pain and fatigue, they were confronted with pain in the feet, cramps, and fatigue in the leg muscles, which constrained them to slow down and make regular stops. When runners were going through challenging environmental conditions (i.e., low temperatures and bad weather conditions), they were also constrained to focus on the difficulties of the terrain to ensure their safety:

Runner: “I really don’t like this descent; it’s really steep, slippery and muddy; it’s the most dangerous.”
Researcher: “So what do you do?”
Runner: “I go really slowly, step after step, I even use my hands. I concentrate on getting through this hard part. Since I just want to finish the race, I don’t care how much time it takes. So I go slowly and I’m really careful.”

When suffering from sleep deprivation and/or hallucinations, runners had to struggle against the onset of fatigue (some of them reported short episodes of hallucinations), which constrained them to improvise small stops to rest:

Runner: “I knew that this pass was hard. But there, I felt slower than usual and, most important, I began to get sleepy. And there I was really sleepy, so I planted my poles, leaned on them and took micro-naps.”
Researcher: “So you made little stops?”
Runner: “That’s right. I leaned on my poles and slept for 2–3 min and then done! I woke up. I must have done it at least 20 times, 20 stops for the same climb; it’s pretty rare for me because usually I’m good at climbing… And then there I say, oh god, this is hard.”

#### Feeling Freedom in the Running Pace

**FIGURE 4:**
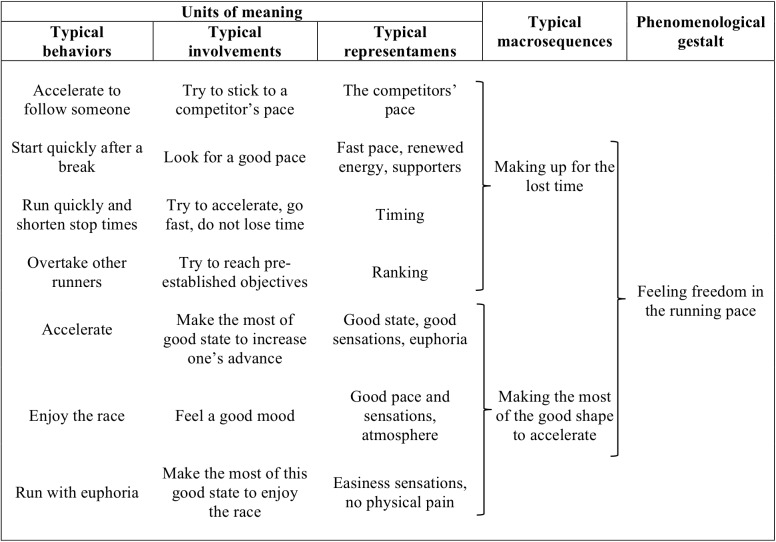
Content of the gestalt: feeling freedom in the running pace.

This phenomenological gestalt refers to the emergence of potential behaviors for going faster, such as setting ambitious time limits, overtaking runners, etc. All these behaviors were associated with positive representamens allowing the emergence of typically free involvements, such as making the most of good states to speed up (Figure 4). This gestalt stemmed from two main concerns: making up for lost time and making the most of feeling in good shape to accelerate. When trying to make up for lost time, the runners reduced the time spent at the refreshment points, and sometimes even decided to skip them in order to keep going along the route. Sometimes the underlying concern was linked to the personal goal of timing and/or the cut-off time. Also, the runners were able to make the most of their actual state to free their pace (i.e., accelerate); they were then able to focus on ranking and/or timing goals:

Runner: “There, I was in a good state because I had just slept, and there I knew that I had to get through the pass to reach Ollomont. But I knew I was in good shape, I was rested, and I had reserves, so I knew very well that I wasn’t going to sleep again before the end of the race. So I said OK, it’s good, I’m tired of waiting, I can speed up now, let’s go.”
Researcher: “So there, on that pass, you are running faster than you did on the other passes?”
Runner: “Yeah. So I get to Ollomont, I ask how long it took the others. The first ones took 4h05 to climb the pass and I took 2h30. So I was doing really good up there! I kept climbing, I checked my altimeter to have an idea about my climbing speed and I was between 800 and 1200 m per hour.”

## Discussion

The aim of this study was to characterize the phenomenological gestalts emerging from trail runners’ activity, which was embedded in the changing situation of an ultra-trail running race. These gestalts revealed three ways of enacting a meaningful world in which situated, embodied, and meaningful processes are entangled to build up trail runners’ activity. The results are original as they outline the multi-textured experience of the lived body in sport (Allen-Collinson, 2009). It is important to keep in mind that our findings revealed that the runners’ experience could not be reduced to one or another of these three gestalts but instead was a combination of them. From this perspective, the runners were able to maintain the enactments of meaningful situations, which were viable for completing the race. These typical worlds emerged from the couplings between their activity and the environment, in association with anticipatory strategies (e.g., training, getting prepared, and elaborating a plan with split times), and naturally led to the emergence of the structure of controlling one’s global ease with an underlying concern for preservation and control. Furthermore, the length of the route, sleep deprivation, or the emergence of pain brought to the fore an experiential structure related to fatigue and the experience of difficult situations. It should also be noted that a participative culture, such as running with peers (e.g., becoming a “finisher” or putting in a performance) may create opportunities for taking risks, which in this case here might have taken the form of freeing oneself from pacing or reducing the time spent resting. Taken together, our results showed how the couplings between the runners and their environment shaped various organizations of their activity; these organizations were graspable at the level of pre-reflexive consciousness and characterized as phenomenological gestalts. They also highlighted the inseparable nature of the emotional, behavioral, and perceptive dimensions of human activity, which, when interacting together, emerge as a meaningful ongoing stream in runners’ experience. In addition, among the singularities in each runner’s experience, we were able to identify similarities in these accounts, suggesting that the runners’ own worlds are shared as they gather similar embodied and cultural experiences; these are entangled in the phenomenological gestalts that will be successively discussed below.

### Discussion of the Phenomenological Gestalts

First, the gestalt of controlling global ease refers to experiencing states in which runners preserve themselves through careful race management. This gestalt is congruent with the findings of Rochat et al. (2017), who showed that finishers spend more time in a state of vitality preservation than withdrawers. It also corresponds to the experience of “ease” identified by Clarke and Haworth (1994). At these moments, the sheer enjoyment of the race and the feeling of personal achievement on becoming a “finisher” seemed to take precedence over concerns of timing and ranking (Simpson et al., 2014); this perspective illutrates one cultural aspect of the ultra-marathon, which is the desire to finish (in contrast to the desire to win), raising a feeling of self-empowerment (Hanold, 2010). In light of goal-setting theory, this observation is congruent with Acevedo et al. (1992), who showed that ultra-marathon runners are more committed to personal performance goals (i.e., finishing the race below a given time) and process goals (e.g., managing efficient breaks, adapting the running technique to the terrain, and splitting the race into shorter steps) than outcome goals (i.e., ranking goal), suggesting that runners are not that concerned with social comparisons. Instead, they tend to set challenging – yet reachable – goals in line with their personal standards (Locke, 1996). Interestingly, Bueno et al. (2008) investigated what they called the “mediator mechanisms” in the efficacy of goal-setting performances in endurance sports. These mediator mechanisms include self-regulatory mechanisms such as self-efficacy, personal goals, threat perception, and coping. Our results suggest that this gestalt of controlling global ease may encompass these self-regulatory mechanisms without, however, partitioning them in a deterministic process (i.e., perceiving the threat, appraising the threat, coping, and performing). From our perspective, self-efficacy and personal goals are co-regulated in the stream of runners’ meaningful activity, and self-regulation may occur before the emergence of the actual threat. Indeed, as runners are aware that they inevitably will have to deal with threatening situations (induced by fatigue onset, weather degradation, etc.), they need to include preservation times in their race management to achieve the goal they are striving for.

When acting in this phenomenological gestalt, the runners had careful running strategies even though some of the procedures were more time-consuming (e.g., sleeping longer, slowing down, etc.). This finding is in line with the observation of a previous study on long-distance walking: once an individual has found a comfortable pace, he/she experiences pleasure and can find sufficient energy to keep going for many hours (Crust et al., 2011). In the same vein, as Johnson et al. (2015) demonstrated, these runners were able to find the balance between positive mood and effort intensity because they were highly self-aware, intrinsically motivated and greatly committed to the challenge. Last, it is important to note that in this gestalt, the runners also adapted to emergent fatigue, yet without feeling negatively impacted or constrained in reaching their personal goals. This observation suggests that fatigue and pain, as well as the prevention of their emergence, is a constitutive part of activity in ultra-trail running, and hence runners do not perceive this kind of situation (i.e., time loss because of breaks or reduced speed) as a threat for reaching their goal. This perspective draws on Hanold’s observation of three levels of pain constructed by ultra-runners: discomfort, good pain and bad pain. She showed that discomfort and good pain are acceptable and normalized by runners, as they are perceived as being inevitable. In addition, Hockey and Allen-Collinson (2016) suggested that runners are familiar with and attuned to a range of normalized sensations, notably because they perform high training mileages. In contrast, bad pain was described as debilitating in the sense that it precludes running and potentially finishing the race (Hanold, 2010). This point on bad sensations is illustrated by the following gestalt.

Second, the gestalt of enduring general fatigue and experiencing difficult situations refers to the difficulties (e.g., health problems, injuries, lack of preparation, or challenging environmental settings) that runners must cope with; it is the experience of surmounting these difficulties with emotional, cognitive, or behavioral strategies, as suggested by research on coping in endurance sports (Evans et al., 2014). Interestingly, Atkinson (2008, 2015) highlighted how triathletes and fell runners come together as a “pain community” and share physical and mental suffering in the sport through a collective quest and self-exploration, which suggests that this typical experience of suffering is embedded in the culture of practice in ultra-endurance sports. However, we also hypothesize that spending too much time in this gestalt might lead to withdrawal (Antonini Philippe et al., 2016). Indeed, runners did not really feel rewarding physical sensations while running during these episodes; the only option they had to reduce their suffering was to take advantage of aid stations to rest. This also helped them to split the race into shorter goals, which is a coping strategy runners often use, as shown by Holt et al. (2014). Furthermore, this difficult state has been described in previous studies that have underlined energy depletion, emotional disturbance, negative energy balance and worsening sleep deprivation throughout the race (Lahart et al., 2013). Moreover, impairments due to lack of sleep (i.e., psychomotor vigilance, increased reaction times, and inability to stay awake), which seem to be recurrent issues during ultra-trail running events (Hurdiel et al., 2015), can be the source of safety issues, mainly for runners who have been struggling for a while. Kruseman et al. (2005) suggested a possible explanation for the lack of energy when they showed that most amateur runners did not meet the energy intake and nutritional needs during a mountain marathon.

Third, feeling freedom in the running pace refers to finding the right attunement to both task constraints and the available organism resources. In this gestalt, the notion of freedom is twofold: On the one hand, it refers to the enaction of a meaningful situation in which runners have more physical and psychological possibilities for undertaking other types of activity than just preserving oneself or coping, such as making up lost time or setting ambitious time limits. On the other hand, freedom is also experienced through rewarding and/or euphoric episodes, which correspond to the experience of flow (e.g., Nakamura and Csikszentmihalyi, 2009) and enable runners to free their activity to make the most of an optimal and (intrinsically) rewarding experience. The state of flow consequently opens opportunities for action that stretches the runners’ existing skills to set and achieve manageable challenges (Nakamura and Csikszentmihalyi, 2014). Also, some runners had episodes of euphoria that can be linked to the expression “runner’s high,” which refers to a rewarding experience resulting from long-distance running. This is associated with the release of endogenous opioids in the frontolimbic brain regions and is correlated with runners’ perceived euphoria (Boecker et al., 2008).

### Contributions to the Enactive Approach

This study makes a twofold contribution to the field of sports psychology: first, it provides a detailed analysis of trail runners’ embedded activity using experience as an entry point without partitioning it. Particularly, our research method expands our access to the dimensions of feelings and the perception of lived experience - and the action and involvement that are linked to them. Second, from a theoretical perspective, the results document the effectiveness of the enactive approach and its underlying 4Es with evidence from the sports science field. Indeed, these three phenomenological gestalts reveal different embodied processes, which emerged in a more or less salient way in the runners’ consciousness as an ongoing stream of experience throughout the race. Even though our results do not present the dynamics of this stream, they revealed the meaningful components of runners’ experience. This conception is therefore congruent with the enactive approach, which considers cognition a process of sense-making of one’s experience (Di Paolo et al., 2011). The various physical sensations (e.g., fatigue, pain, injuries, euphoria, ease, etc.) support the assumptions that activity is embodied and that significant embodied elements make sense to runners as they try to organize their activity to maintain physical safety by taking these elements into account. For example, physical alerts are significant elements, signals that help runners to involve in the situation more carefully. As a further perspective and in congruence with the sense-making process, a deeper analysis of how the physical alerts are perceived and interpreted by runners may be interesting for understanding how they develop a “haptic knowing” about their activity (Allen-Collinson and Hockey, 2011, p. 341). These embodied elements are linked with the embedded dimension of their activity in the sense that bad conditions affect them physically, leading to health issues (cold, injuries, etc.), and also psychologically, affecting morale. To cope with uncomfortable sensations, runners may turn to equipment as an extended dimension of activity. For example, poles can be used to sleep on and waterproof jackets are affordances when the situation requires adaptations (Seifert et al., 2014). From this perspective, the runners enacted a meaningful world from the environmental contingencies and made adaptations, and these processes contribute to explaining how performance is achieved.

### Limitations of the Study

This study had limitations. First, our analysis did not determine the interactions between the participants’ gender and/or level of performance and expertise and the present results. Second, we did not quantify the duration of the gestalts, nor did we restore their temporal layout in the runners’ experience; we do, however, offer a perspective for conducting further research to investigate whether typical activity profiles are identifiable. Last, the retrospective design was a limitation because the enactive interview was a new situation and this raised the risk that the runners would build new meanings regarding past experience. This issue is especially important for ultra-endurance racing as some runners had lived hallucinations and losses of lucidity, raising questions about account accuracy due to blurred memories in certain race sections, and about the lived temporality of events, especially in critical situations. In the same vein, the retrospective design raises a question about the role of the result (i.e., personal time and/or ranking) on the expressions of one’s experience: we may hypothesize that runners who were disappointed by their performance were more likely to provide more negative accounts/narratives of their race. However, these issues linked to the retrospective design were noted by Hauw and Bilard (2012) and Hauw and Mohamed (2015), who used similar interview techniques, but these authors found that the limitation could be reduced by providing traces to help the runners re-enact their past experience and asking them to ignore the outcomes. Therefore, this new situation (i.e., the re-enactment of the past experience induced by the interview techniques) was ultimately quite similar to the lived experience in the race situation, providing us an acceptable level of quality and authenticity of the accounts; this perspective has already been addressed in previous studies in trail running, which used a similar methodology for data collection (i.e., Antonini Philippe et al., 2016; Hauw et al., 2017; Rochat et al., 2017).

### Proposition of Practical Recommendations

It has been shown that preparing for ultra-events relies heavily on self-regulating training practices (Krouse et al., 2011), and our results suggest some practical applications especially for non-professional runners who seek to develop a viable practice of ultra-trail running. We will first outline the main practical recommendations provided by prior studies in trail running and then propose some additional recommendations based on our results.

According to the general recommendations (e.g., Simpson et al., 2014), important decisions should be made before the race and then become a resource plan for adjusting activity (Suchman, 2006). This perspective suggests that during the preparation period, setting a tactical plan (e.g., estimating lap times, splits, etc.) and learning about the trail will help race management and ultimate performance: Scouting the route ahead of time will enhance runners’ knowledge of the terrain and prepare them to adapt their pace in function of the different route segments, thereby increasing tactical efficiency. Organization and preparation are important components of trail running activity (Simpson et al., 2014), and strategy is a key element for completing a race. While managing the race, runners should maintain physical integrity by delaying the onset of muscular pain and using the feeling of physical easiness and comfort as signals for controlling the pace and procedures (Rochat et al., 2017). Hurdiel et al. (2015) suggested that runners should actively manage sleep with a predefined plan based on the race duration.

In the light of our results, we suggest additional recommendations to complete the above-mentioned range of strategies. Other steps to include might be: lengthening the sleep period to avoid losses in lucidity and/or extreme exhaustion states, making the most of the social environment by sitting with friends and family at the aid stations, sticking to a slower pace, and finding other runners as social support. Our results also highlighted that runners need periods in the race during which they run alone at their own pace, opening the possibility to stay mindful of their own physical sensations. A way to efficiently combine the emergence of bad physical sensations and the need for adaptation to environmental conditions would be – when possible – resting when the meteorological conditions are poor. Last, the enactive interviews could be used for intervention to (1) improve athletes’ follow-up for sports psychologists, (2) invite reflexive practice on one’s own activity to develop a better understanding of performance by reliving one’s past experience (Hauw, 2009, 2018), and (3) investigate the suffering episodes and critical situations in greater detail by reducing the granularity of analysis using micro-phenomenological interviews (e.g., Depraz et al., 2017). In doing so, we may expect that runners are able to connect these typical gestalts to their own personal experiences and stories as trail runners in order to sustain an autonomous, responsible, and self-regulated approach to their sport.

## Author Contributions

NR, VG, and DH collected and processed the data. NR, VG, LS, and DH co-wrote the manuscript.

## Conflict of Interest Statement

At the time when the study was conducted, NR was employed by Raidlight-Vertical Outdoor Lab Company, Saint-Pierre-de-Chartreuse, France, and was funded by a grant from the ANRT (Association Nationale Recherche Technologie) under a CIFRE agreement (Industrial Convention of Learning by Research) with Raidlight and Swiss universities. The funder provided support in the form of a salary for the author but did not have any additional role in the study design, data collection and analysis, decision to publish, or preparation of the manuscript. This commercial affiliation does not alter our adherence to all Frontiers policies on sharing data and materials. This research was also suppported by the contract plan State – Region (CPER) with the ID: Smart Mobility, Logistic, Numeric (XTerM). The remaining authors declare that the research was conducted in the absence of any commercial or financial relationships that could be construed as a potential conflict of interest.
