# Supplementary figures and images for: Enacting Phenomenological Gestalts in Ultra-Trail Running: An Inductive Analysis of Trail Runners’ Courses of Experience
Source: Front Psychol. 2018 Oct 26;9:2038. doi: 10.3389/fpsyg.2018.02038 (PMC6213919; doi:10.3389/fpsyg.2018.02038)

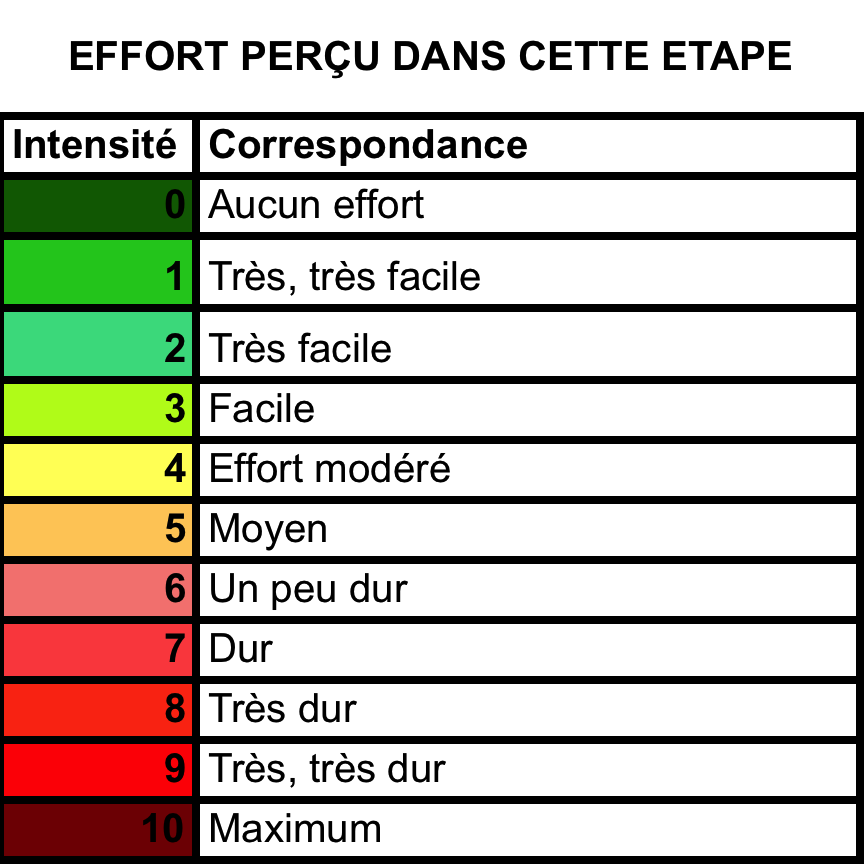


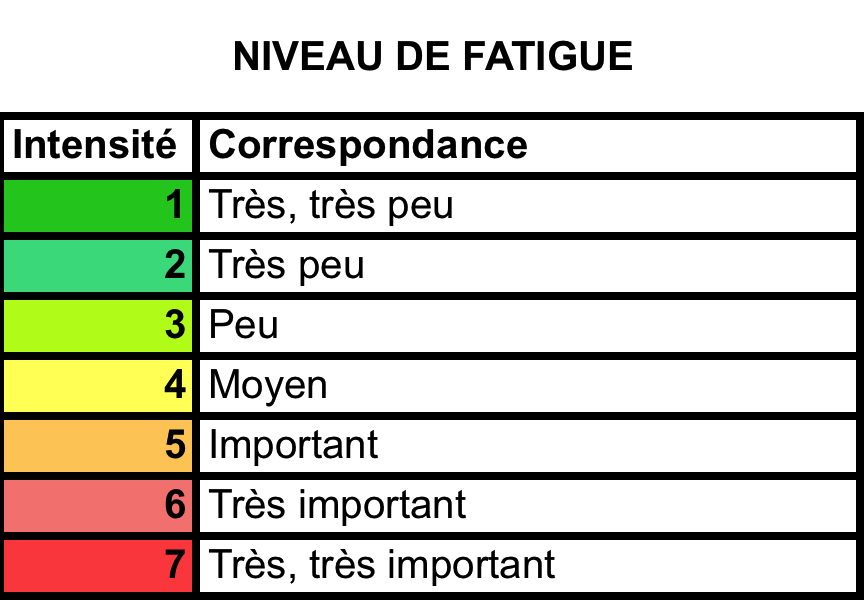


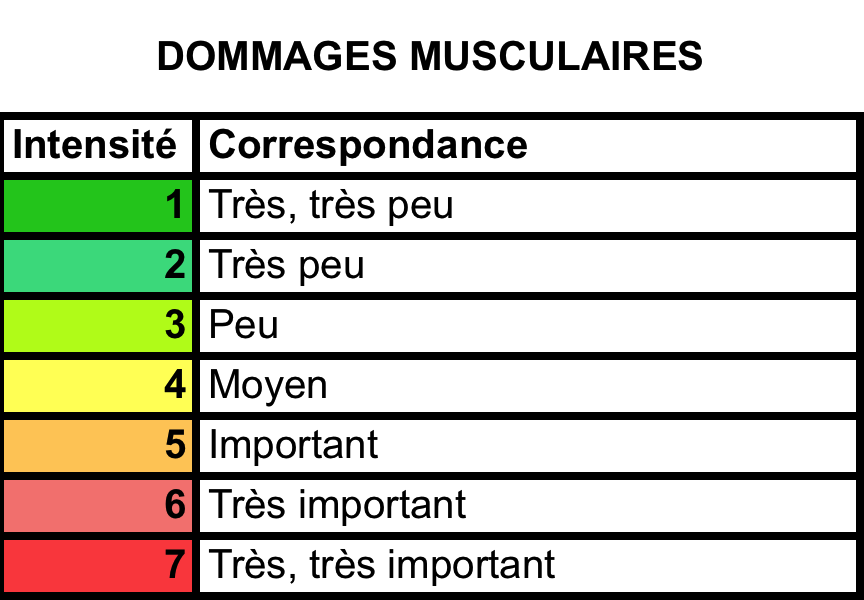


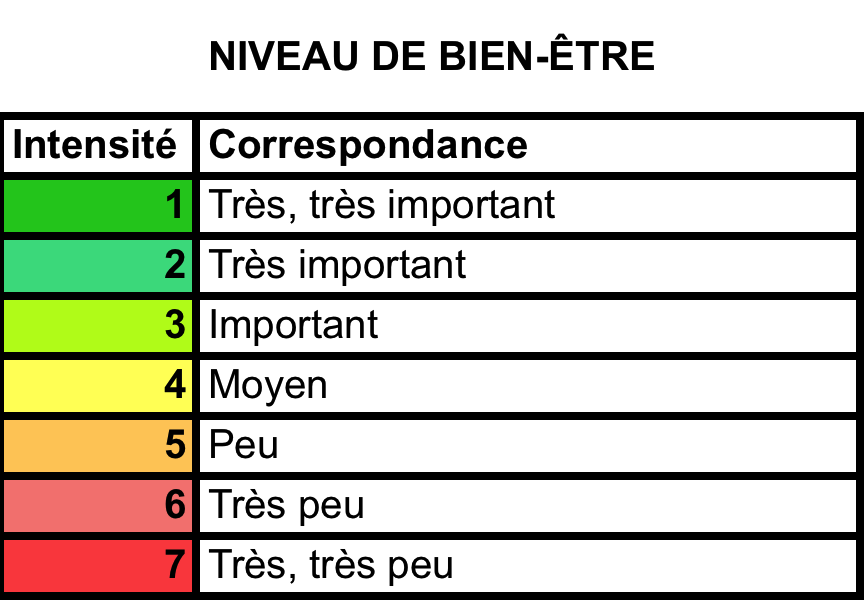

Supplement: Supplementary file 1 [file Table_1.DOCX]
